# An interpretable rule-based diagnostic classification of diabetic nephropathy among type 2 diabetes patients

**DOI:** 10.1186/1471-2105-16-S1-S5

**Published:** 2015-01-21

**Authors:** Guan-Mau Huang, Kai-Yao Huang, Tzong-Yi Lee, Julia Tzu-Ya Weng

**Affiliations:** 1 Department of Computer Science and Engineering, Yuan Ze University, Chung-Li, Taiwan; 2 Innovation Center for Big Data and Digital Convergence, Yuan Ze University, Chung-Li, Taiwan

## Abstract

**Background:**

The prevalence of type 2 diabetes is increasing at an alarming rate. Various complications are associated with type 2 diabetes, with diabetic nephropathy being the leading cause of renal failure among diabetics. Often, when patients are diagnosed with diabetic nephropathy, their renal functions have already been significantly damaged. Therefore, a risk prediction tool may be beneficial for the implementation of early treatment and prevention.

**Results:**

In the present study, we developed a decision tree-based model integrating genetic and clinical features in a gender-specific classification for the identification of diabetic nephropathy among type 2 diabetic patients. Clinical and genotyping data were obtained from a previous genetic association study involving 345 type 2 diabetic patients (185 with diabetic nephropathy and 160 without diabetic nephropathy). Using a five-fold cross-validation approach, the performance of using clinical or genetic features alone in various classifiers (decision tree, random forest, Naïve Bayes, and support vector machine) was compared with that of utilizing a combination of attributes. The inclusion of genetic features and the implementation of an additional gender-based rule yielded better classification results.

**Conclusions:**

The current model supports the notion that genes and gender are contributing factors of diabetic nephropathy. Further refinement of the proposed approach has the potential to facilitate the early identification of diabetic nephropathy and the development of more efficient treatment in a clinical setting.

## Background

Diabetes is a metabolic disorder characterized by the inability of the pancreas to produce enough insulin or the body's lack of ability to effectively use insulin. The disease is contributed by multiple factors, including diet, lifestyle, and genes. Despite the advancement in healthcare, the prevalence of diabetes is still on the rise. More than 150 million people worldwide are affected with this debilitating disease [1]. Thus, the surveillance, prevention, and control of diabetes and its complications are becoming increasingly important.

Diabetes can result in various complications, damaging the heart, blood vessels, eyes, kidneys, and nerves. As one of the major microvascular complications of diabetes, diabetic nephropathy affects about 30% of the people with type 1 diabetes (T1D), and 25-40% of the people with type 2 diabetes (T2D) [2]. DN is characterized by the persistent elevated levels of albumin in the urine, progressive decline in the glomerular filtration rate, and increased arterial blood pressure. In fact, DN is among the leading causes of end stage renal disease (ESRD), imposing serious impact on morbidity, mortality and the patients' quality of life [2]. Compared to non-diabetics, the likelihood of dying from renal disease is 17 times greater for diabetics [3].

Generally, DN symptoms are not obvious. In T1D patients, DN develops over an initial 10 to 15 years of diabetes; whereas in T2D patients, the onset is less clearly defined [4]. When clinical indices for renal functions (e.g. urinary protein level) become abnormal, the kidneys have already been significantly damaged and prevention against ESRD may be too late at this stage [5]. Thus, it would be beneficial to develop a prediction model utilizing various clinical measures, such as gender, history of diabetes, body mass index (BMI), etc. In addition, since existing studies suggest that genes and gender play specific and significant roles in DN [3,6,7], integrating genetic and gender information in the prediction of DN susceptibility may lead to more effective prevention or treatment.

To date, two groups have attempted to construct prediction models for DN. Cho et al. (2007) utilized a support vector machine (SVM) approach to classify DN patients among type 2 diabetics, but the model was trained on an irregular and unbalanced dataset [8]. On the other hand, Leung et al. compared various machine learning and statistical methods to develop a prediction strategy for the identifcation of genotype-phenotype risk patterns in DN [9]. Combining genotype and clinical information as features for classification through SVM and random forest approaches was found to generate the best performance [9]. However, as diabetes and DN are caused by multiple factors [1,3,7], a model designed for one community may not be applicable to another. Moreover, prediction results generated from computational algorithms may be difficult for clinicians to explain to patients regarding their risk of developing DN and convince the patients to take the necessary preventative measures. Therefore, a straightforward and user-friendly tool is more practical in a clinical setting.

Previously, Wu et al. [10,11] have conducted a candidate gene analysis on 345 T2D patients, analyzing the association of 20 candidate genes with T2D, as well as the related complications such as obesity and DN. Through the generalized multi-dimensional reduction approach, Wu et al. have identified various gene-gene interactions that may represent genetic susceptibilities to T2D, obesity, and DN [10,11]. The findings suggest that T2D and DN may be attributable to multiple factors that include the interactions between specific genes and the environment.

In the present study, we employed a gender-based rule in a decision tree approach to identify the risk for DN among T2D patients using the data provided by Wu et al [10,11]. Similar to Leung et al.'s finding (2013), our results indicate that the integration of clinical and genetic features yielded better performance in distinguishing DN from non-DN diabetic patients [9]. Furthermore, the nature of decision tree classification may help simplify interpretation of the results. This easy-to-understand prediction tool may be beneficial for the early detection of T2D patients susceptible to DN.

## Results

### Performance comparison between individual and combinations of clinical features

The performance of using individual clinical features for DN classification analysis is shown in Tables S3-S12. Among the clinical attributes, blood creatinine (BC), blood urinary nitrogen (BUN), and urinary albumin were the three renal indices that generate the best performance (Table 1). In contrast, other clinical features that do not directly reflect kidney functions, including serum triglyeride level and high-density lipoprotein (HDL) level, were much lower in accuracy, specificity, and sensitivity. For the purpose of prediction, we decided to exclude direct kidney function indicators for subsequent analyses (Additional file 2: S3-S12).

**Table 1:** The top five best performing clinical features for diabetic nephropathy classification.

Feature	Decision tree	Random forest	SVM	Naïve Bayes
**Urinary albumin**	96.23%	95.36%	95.49%	95.13%
**BUN**	85.51%	82.90%	82.61%	79.57%
**BC**	83.19%	80%	84.72%	81.30%
**Serum triglyceride**	60.87%	56.53%	57.89%	59.57%
**HDL**	63.91%	62.32%	59.13%	55.22%

In order to assess whether feature selection would enhance the classification performance, each features were evaluated based on their information gain (Table S7) or F-scores (Table S8). Table 2 shows the classification performance of implementing variable numbers of the top ranking features in different classifiers. The best accuracy, sensitivity, and specificity were achieved by the three-, six-, and seven-attribute model in SVM, random forest, and Naïve Bayes, as well as decision tree, respectively. Overall, the performance was slightly better than using individual clinical features for classification, but it was still much lower compared to that of BC, BUN, or urinary albumin.

**Table 2:** Performance of utilizing variable numbers of clinical features in different classifiers to predict diabetic nephropathy.

Classifier	No. of features	Accuracy (%)	Sensitivity (%)	Specificity (%)
Decision tree	7	62.17	55.56	69.02
Random forest	6	63.91	60.68	67.26
SVM	3	60.87	50	78.72
Naïve Bayes	7	62.61	41.03	84.96

### Performance comparison between individual and combinations of genetic features

We followed the same procedures to evaluate the possibility of employing genotype information from the 20 candidate genes in DN classification. Compared to the clinical attributes, individual genetic features performed poorly, with accuracy, specificity, and sensitivity measures ranging from 48% to 57%, 36% to 100%, and 0% to 69%, respectively (Tables 3 and S13-S16). The result was similar when we attempted to select the best features by information gain and F-score analyses (Table S17 and S18), and compared the performance of using various top-ranking attributes in different classifiers (Tables 4 and S19-S22). The best performance was achieved when four, 12, and 13 genetic features were implemented to construct the prediction model via decision tree, random forest, and SVM, as well as Naïve Bayes, respectively. Nevertheless, similar to using various combinations of clinical features for classification, the improvement in performance was marginal (Additional file 3: S13-S22).

**Table 3:** The top five best performing genetic features for diabetic nephropathy classification.

Feature/Classifier	Decision tree	Random forest	SVM	Naïve Bayes
**FTO**	57.39%	51.01%	47.83%	56.52%
**ENPP1**	55.22%	53.91%	49.13%	55.22%
**ADIPOQ**	53.48%	53.04%	55.22%	53.48%
**GHSR (rs9819506)**	53.48%	53.04%	47.83%	53.48%
**GHSR (rs490683)**	53.91%	53.04%	47.52%	52.17%

The GHSR gene is represented by two SNP IDs.

**Table 4:** Performance of utilizing variable numbers of genetic features in different classifiers to predict diabetic nephropathy.

Classifier	No. of features	Accuracy (%)	Sensitivity (%)	Specificity (%)
Decision tree	4	60.43	54.70	66.37
Random forest	12	53.91	59.83	47.79
SVM	13	53.04	67.65	31.9
Naïve Bayes	13	56.09	58.12	53.98

### Performance evaluation on the integration of clinical and genetic features for DN classification

Perhaps by looking at clinical and genetic features separately, we have ignored the possible interactions among genes and clinical traits that underlie the disease mechanisms of DN. Thus, we tried integrating both clinical and genetic attributes for classification. First, the best features were selected based on their corresponding information gain and F-scores (Table S23 and S24). Then, the top ranking clinical and genetic features were integrated for performance evaluation in different classifiers. Overall, a slight increase in performance was observed (Tables 5 and Additional file 4: S23-S28)

**Table 5:** Performance of utilizing variable numbers of genetic and clinical features to predict diabetic nephropathy.

Classifier	No. of features	Accuracy (%)	Sensitivity (%)	Specificity (%)
Decision tree	7	65.22	63.25	67.26
Random forest	25	65.09	60.68	71.68
SVM	7	61.23	66.17	51.06
Naïve Bayes	10	63.91	46.15	82.30

In particular, when 25 of the 32 features were utilized for classification, random forest appeared to generate the best results. Decision tree seemed to be the second best classifier, when features including serum triglyceride, ADRB2, ENPP1 (ectonucleotide pyrophosphatase/phosphodiesterase 1), gender, fasting plasma glucose, TCF7L2 (transcription factor 7-like 2), ADIPOQ (adiponectin, C1Q and collagen domain containing protein) were employed for classification. This approach appeared to outperform previous attempts using clinical or genetic features separately. Yet, the classification performance was still much lower compared to renal function markers such as urinary albumin, BC and BUN (Table 1).

### Optimization of DN classification performance by the implementation of a gender-based rule

Based on these results, we decided that decision tree and random forest worked best with our data. However, our objective was to construct an interpretable classification model that can be easily applied in a clinical setting. In this case, decision tree holds an advantage over random forest as the former classifies individuals among a range of numerical and categorical features, following certain set rules in a way that is similar to human decision making. The latter, on the other hand, is not as easy to interpret because trees are arbitrarily added to the forest. Therefore, we focused on using decision tree for subsequent classification attempts.

Since genes and gender are known contributing factors to DN risk [3,6,7], we postulated that the features required to discriminate DN among T2D patients may be different between males and females. Thus, we implemented a gender-based rule, separating the training data based on gender prior to classification. When applied on the training data and assessed with a five-fold cross validation approach, the proposed method yielded better classification results with the decision tree model (Table 6), with accuracy, specificity, and sensitivity reaching 85.27%, 83.32 and 85.24%, respectively. On a separate testing dataset (Table 7), the classification accuracy, specificity, and sensitivity were 78.50%, 80.64 and 81.40%, respectively. Performance evaluation of the other classifiers are shown in Tables S29-S31. Note that, for males with more than 17 years of diabetic history, none of the features, except for urinary albumin, could accurately predict DN (Additional file 5: S29-S31).

**Table 6:** Performance of gender-based decision tree classification of diabetic nephropathy in the training dataset.

Group	Feature	SN(%)	SP(%)	ACC(%)
**1. Female, BMI < 24**	Serum triglyceride Fasting plasma glucose IGF2BP2	81.82	100	93.10
**2. Female, BMI > 24**	HDL LDL ENPP1 PCSK1	76.27	72.88	74.58
**3. Male, history<11(years)**	Serum triglyceride LDL UCP2 FTO	73.68	74.47	74.24
**4. Male, 11<history<17(years)**	Serum triglyceride Fasting plasma glucose ADIPOQ	97.72	69.23	87.14
**5. Male, history>17(years)**	Urinary albumin	96.88	100	97.06

**Table 7:** Performance of gender-based decision tree classification of diabetic nephropathy in the testing dataset.

Group	Feature	SN(%)	SP(%)	ACC(%)
**1. Female, BMI < 24**	Serum triglyceride Fasting plasma glucose IGF2BP2	75	90.91	84.24
**2. Female, BMI > 24**	HDL LDL ENPP1 PCSK1	69.23	73.08	71.79
**3. Male, history<11(years)**	Serum triglyceride LDL UCP2 FTO	63.63	69.23	72.73
**4. Male, 11<history<17(years)**	Serum triglyceride Fasting plasma glucose ADIPOQ	84.62	70	78.26
**5. Male, history>17(years)**	Urinary albumin	100	100	100

**Figure 1:**
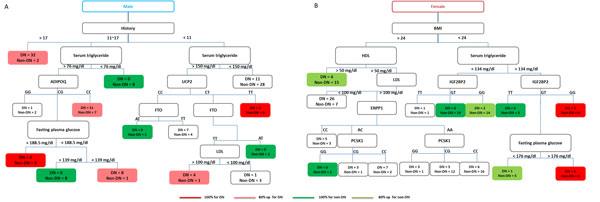
**Decision tree classification of diabetic nephropathy among male and female type 2 diabetics**. Accuracy measures are represented by the color intensity. Red indicates 100% prediction accuracy for DN. Pink indicates a prediction accuray above 80% but below 100% for DN. Green indicates 100% prediction accuracy for non-DN. Light green indicates a prediction accruacy above 80% but below 100% for non-DN.

The gender-based classification approach resulted in substantially enhanced performance compared to using individual clinical features, genetic features, or the combination of these two types of attributes. Figures 1A and 1B show the way that male and female diabetic patients would be classified in our decision tree model for DN identification. Red and green colors indicate DN and non-DN, respectively. Accuracy measures are represented by the intensity of the colors.

Although the performance of the proposed gender-based strategy may not exceed that of renal function markers, the model presents an easily interpretable classification tree for DN prediction. For example, in Figure 1B, a female patient with BMI less than 24 and serum triglyceride level greater than 134 mg/dl, may very likely be susceptible to DN if she exhibits a GG genotype on the IGF2BP2 (insulin-like growth factor 2 mRNA binding protein 2) gene. In contrast, if she possesses the TT genotype, her possibility of developing DN is relatively low. However, if she is heterozygous at this locus (i.e. the GT genotype), the next determining factor of whether or not she belongs to the high-risk group would be her fasting plasma glucose level. Thus, the constructed model may make it easier for clinicians to efficiently identify DN risk among T2D patients based on their clinical examination results as well as their genetic susceptibility.

## Discussion

Multiple environmental and genetic factors affect the disease progression of diabetes and its associated kidney complications [3,7,12], leading to various chronic and complex health problems. The aim of the present study was to develop a systematic risk assessment model that aid clinicians in the identification of DN patients to faciliate effective monitoring and efficient use of medical resources. As the purpose was to assess the possibility of early prevention, parameters directly associated with renal functions, such as blood creatinine (BC), blood urinary nitrogen (BUN), and urinary albumin, were removed. Under these conditions, our study focused on analyzing the performance of utilizing various clinical (excluding direct renal function markers) and genetic attributes, as well as the integration of a gender-based rule in a decision tree to classify DN patients in a T2D dataset.

Our present finding supports the notion that mechanisms underlying DN may be attributable to multiple genetic and environmental factors. When individual genetic or clinical attributes were employed in the classification of DN patients from the T2D data, the performance was, however, disappointing. A slight increase in performance was achieved after the clinical and genetic features were combined for classification. The results correspond with existing observations that each genetic and environmental determinant may contribute moderate effects to DN, but when interacting together, these factors may significantly influence the progression of DN in T2D patients [13].

Therefore, when making predictions on the development of DN for T2D patients, it may be important to consider both genetic and clinical factors. In fact, many chronic disease-related studies also agree on the integration of clinical and genetic parameters in making better predictions about disease risks [9,14,15]. For complex diseases like T2D and its associated DN complication that are genetically heterogeneous, the same genetic features that may help predict DN susceptibility for one patient may not apply to everyone. However, classifying individuals based on clinical and genetic differences may present to be a logical solution.

Unlike existing models [8,9], we implemented a gender-based rule in the decision tree classification of DN. The SVM prediction tool introduced by Cho et al. [8] was built on unbalanced datasets. While Leung et al.'s model achieved almost 100% accuracy in the prediction of T2D kidney diseases as a result of training their model on a much bigger sample size [9], their tool is also derived from SVM. Despite being the most effective at handling numeric data, the result of an SVM analysis is not necessarily "clinician or patient friendly," potentially inhibiting it from being widely applied in clinical settings.

In contrast, the simplicity of decision tree learning, in addition to its ability to handle both non-homogeneous and nonlinear data in common clinical situations where outliers, missing attribute values, or misclassified points exist, becomes an advantage [16]. Moreover, the results generated by decision tree-based methods are easy to interpret as the approach discovers if-then rules from a given dataset. Most of these merits may not be easily attainable by conventional classification methods such as linear discrimination analysis or k-nearest neighbor method. Although our attempts on classifying DN based on the combination of clinical and genetic parameters failed to outperform common renal funtion indices, by implementing a gender-based rule which follows existing observations that DN risk differs between males and females [7,12,17], we were able to increase the performance of DN identification significantly.

Our approach provides additional support for the emerging evidence that DN risk is gender-specific. For example, for female patients, BMI, serum triglyceride level, fasting plasma glucose level, the IGF2BP2 gene were the features that best discrminate DN from non-DN individuals. It is known that the level of triglycerides is higher in DN patients with T1D [18] and T2D [19]. In addition, the IGF2BP2 gene has been associated with DN in males with T1D [20] and appears to modulate the risk of T2D among obese individuals of the Chinese Han population [21]. Although, currently, there is insufficient evidence correlating the IGF2BP2 gene with DN in T2D females, our model suggests that female patients with the TT genotype at the IGF2BP2 gene locus may be at a lower risk of developing DN compared to those with the GG genotype.

Likewise, according to our analysis, the primary determining factors for the development of DN for male T2D patients lie in the duration of T2D and serum triglyceride level. Different genes appear to act on the risk of DN for males with different durations of T2D. For instance, males with 11 to 17 years of T2D history, would very likely develop DN if they have moderately high levels of serum triglyceride and possess the CC genotype at the ADIPOQ gene locus. In contrast, those heterozygous at this gene locus must exhibit high fasting plasma glucose levels to have increased risk of developing DN. Though the ADIPOQ gene has been associated with T2D [22,23] and DN [11], the exact nature of this association has not been identified. Our results imply that the ADIPOQ gene may exert its effect on DN risk among individuals with specific clinical attribute values. In addition, our model suggests that for males that have lived with T2D for less than 11 years, if they are heterozygous at the obesity and T2D candidate gene, FTO (fat mass and obesity associated) [24], they are more likely to develop DN when their LDL levels exceed 100 mg/dl.

## Conclusions

To our knowledge, as the disease mechanisms of DN are complex and the symptoms are not obvious, there are no accurate and easy-to-interpret diagnostic methods for the early screening of DN susceptibility. By performing a series of decision tree analyses with the integration of a gender-based rule, we have identified specific clinical and genetic features, as well as their hidden associations and interactions, that may be used for DN risk assessment. The proposed strategy generates results that are easy-to-interpret and easy-to-implement. With further refinement in parameter settings and testing in a bigger sample size, our approach may have the potential to facilitate early identification of individuals with DN susceptibility and therefore, efficient prevention or treatment for DN.

## Methods

### Participants

The T2D data were kindly provided by Dr. L.S.H. Wu. The data consisted of 345 Taiwanese patients recruited from the Tri-Service General Hospital in Taipei, Taiwan, in 2002. These data were obtained in previously published studies under the approval of the insititutional review board at the the Tri-Service General Hospital Taipei,Taiwan. The criteria for recruitment has been described in Wu et al. [10,11]. All recruited patients fulfilled the following criteria: (1) diagnosed with diabetes for >5 years; (2) age 30 to 75 years; (3) The fasting plasma glucose was >6.93 mmol/l (126 mg/dl); (4) The glycated haemoglobin (HbA1C) was >6%. Patients who were classified as the DN group fulfilled any of the following three criteria: (1) the average ACR was >300 μg/mg; (2) the BUN was >20 mg/dl; and (3) the serum creatinine was >1.7 mg/dl. The rest of the patients were classified as the T2D without DN group. The case group comprises 185 T2D patients with DN, and the control group comprised 160 T2D without DN. Table S1 (Additional file 1) provides an differences in demographic and clinical characteristics among the participants were assessed via Student's t-test. The p-value < 0.05 was regarded as statistically significant. In Wu et al [10,11], genotypes on the 20 T2D candidate genes have already been determined, and the genotype distribution for each gene is shown in Table S2 (Additional file 1).

### System flow

**Figure 2:**
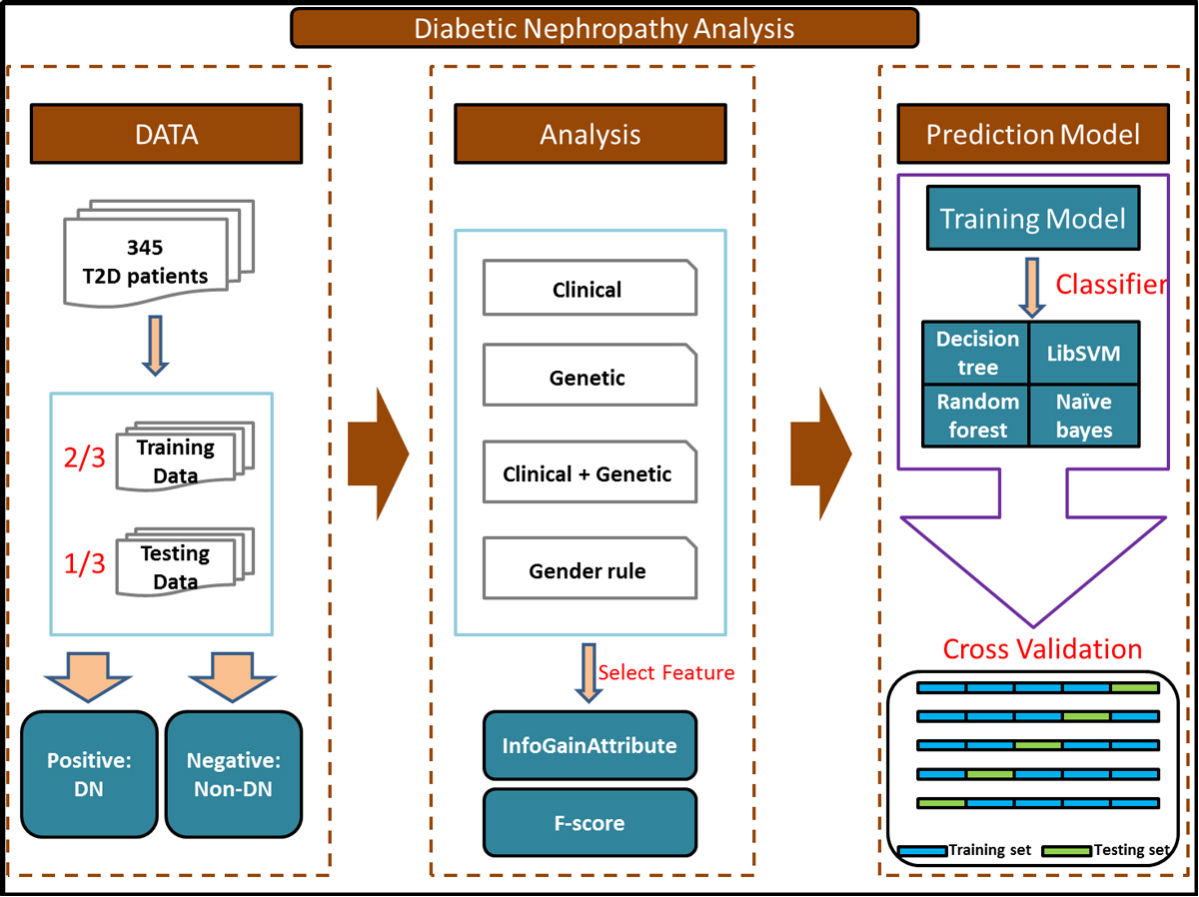
**System flow of diabetic nephropathy analysis**.

The system flow of our work is illustrated in Figure 2. Our data were divided into three parts: 2/3 as the training and 1/3 as the testing dataset. The training dataset was composed of 117 positive and 113 negative data, while the testing dataset consisted of 68 positive and 47 negative data. Features were selected based on their corresponding information gain scores for decision tree and naïve Bayes, or F-scores for SVM. A five-fold cross-validation analysis was conducted to assess the performance of the following strategies in classifying DN by the decision tree, random forest, libsvm and naïve Bayes approaches: 1) selected clinical features only, 2) selected genetic features only, 3) combinations of clinical and genetic features, 4) a gender-based rule integrated with selected clinical and genetic features.

### Feature selection

Experiments were conducted in LibSVM (version 3.12) [25] and WEKA, or Waikato Environment for Knowledge Analysis (version 3.6.5) [26], a JAVA based platform for data mining and data analysis. Since diabetes and its associated complications can be caused by multiple factors [12,17], the best features from our dataset for DN classification were identified by computing the F-scores in LibSVM for SVM, and the information gain scores via the InfoGainAttribute in WEKA [27] for decision tree and naïve Bayes. Features were subsequently ranked based on these scores.

### F-score

F-score [28] is a feature selection technique that measures the extent by which a certain attribute can discriminate a dataset into different classes. Given training vectors *x_k_*, k = 1,2,3...,m, if *n*_- _and *n*_+ _denote the number of positive and negative instances, respectively, then the F -score of the *i *th feature can be calculated by the following formula.

$$\text{F}\left(\text{i}\right)=\frac{{\left({{\bar{x}}_{i}}^{\left(+\right)}-{\bar{x}}_{i}\right)}^{2}+{\left({{\bar{x}}_{i}}^{\left(-\right)}-{\bar{x}}_{i}\right)}^{2}}{\frac{1}{n_{+}-1}\sum_{k=1}^{n_{+}}{\left({x_{k,i}}^{\left(+\right)}-{{\bar{x}}_{i}}^{\left(+\right)}\right)}^{2}+\frac{1}{n_{-}-1}\sum_{k=1}^{n_{-}}{\left({x_{k,i}}^{\left(-\right)}-{{\bar{x}}_{i}}^{\left(-\right)}\right)}^{2}}$$

where the average of the *i*^th ^feature of the whole, positive, and negative datasets are represented by ${\bar{x}}_{i},{{\bar{x}}_{i}}^{\left(+\right)},{{\bar{x}}_{i}}^{\left(-\right)}$, respectively. $x_{k,i}^{\left(+\right)}$indicates the *i*-th feature of the *k- *th positive instance, while $x_{k,i}^{\left(-\right)}$ is the *i*th feature of the *k*th negative instance. Features with larger *F*-scores are more discriminative.

### Information gain

The InfoGainAttribute tool in WEKA presents a simple feature selection algorithm that evaluates the ability of an attribute in making accurate and specific classification in a dataset by measuring its information gain according to the following formula:

$$IG\left(A,S\right)=H\left(S\right)-\underset{t\in T}{\sum }p\left(t\right)H\left(t\right)$$

where information gain IG(A) is the measure of the difference in entropy from before to after the dataset S is split by an attribute A, and t represents the subsets created from the splitting, such that $\text{S}={⋃}_{t\in T}t$, and p(t) is the proportion of the number of elements in t to the number of elements in dataset S. H is the information entropy, which is calculated according to the following formula:

$$H\left(S\right)=-\underset{x\in X}{\sum }p\left(x\right){\text{log}}_{2}p\left(x\right)$$

where S is the dataset for which entropy is being calculated, × represents the classes in S, and p(x) is the proportion of the number of elements in class × to the number of elements in set S.

### Decision tree learning algorithm

Next, we used the C4.5 decision tree algorithm [29] in WEKA to perform DN classfication. The C4.5 algorithm incorporates the concept of information gain to generate the best tree for classification and has become a standard learning tool for the supervised classification problem domain. C4.5 is an extension of the ID3 decision tree induction algorithm [30] with additional features, including the ability to manage continuous attribute values and noise, deriving alternative measures for selecting attributes, and pruning tees [29]. Tree induction in C4.5 involves the following three steps [31]: 1) construction of a large tree from the training data according to attribute selection by information gain scores; 2) removal of branches to avoid overfitting; 3) processing of the pruned tree to enhance its interpretability. The performance of each generated tree was evaluated by five-fold cross-validation.

### SVM

SVM is a machine learning algorithm which attempts to find a hyperplane that best differentiate the data into one category or the other [32]. It is often used for classification, regression, and other learning tasks. We selected the features according to their corresponding F-scores and performed classification based on the higher ranking features via SVM in LIBSVM [25].

### Naïve Bayes

Naïve Bayes, coupled with information gain analysis as the feature selection method, was evaluated for its performance in classifying DN and non-DN patients in the T2D dataset. Naïve Bayes [33,34] is generally used for text classification due to its computational efficiency and relatively good predictive performance. The formula of Naïve Bayes follows Baye's theorem of probability:

$$\text{P}\left(C_{k}|x\right)=P\left(C_{k}\right)\times \frac{P\left(x|C_{k}\right)}{P\left(x\right)}$$

where P(*C *= *C_k_*|*X *= *x*) is the probability that an item belongs to class *C_k_*, given that C_k _has a feature vector x. Having made clear that *C_k _*and × are values taken on by random variables C and × simplify notation by omitting those random variables. For instance, the expected number of classification errors can be minimized by assigning a document with feature vector × to the class *C_k _*for which P(*C_k_*|*x*) is high.

### Random forest

Random forest was also included in our study as one of the classifiers for building the DN prediction model. The random forest algorithm [35] outputs a collection of decision trees based on the random selection of features. Individual trees may exhibit specific prediction errors. However, when aggregated, it is expected that each tree can capture different patterns within the data and generate better performance [36].

## Competing interests

The authors declare that they have no competing interests.

## Authors' contributions

GMH and JTYW conceived the experimental design and wrote the manuscript. GMH performed the necessary analyses to construct the classification model. TYL and KYH participated in the experimental design and coordination of the project. All authors read and approved the final manuscript.

## Supplementary Material

Additional file 2 Table S3-S12: **Performance of individual or selected clinical features for diabetic nephropathy classification**. This file can be viewed with: **Microsoft Excel Viewer Table S3**. Performance of individual clinical features for diabetic nephropathy classification via decision tree. **Table S4**. Performance of individual clinical features for diabetic nephropathy classification via random forest **Table S5**. Performance of individual clinical features for diabetic nephropathy classification via SVM. **Table S6**. Performance of individual clinical features for diabetic nephropathy classification via Naïve Bayes. **Table S7**. Ranking of the 12 clinical features based on their information gain scores **Table S8**. Ranking of the 12 clinical features based on their F-scores **Table S9**. Performance of using variable numbers of clinical features to predict diabetic nephropathy via decision tree. **Table S10**. Performance of using variable numbers of clinical features to predict diabetic nephropathy via random forest. **Table S11**. Performance of using variable numbers of clinical features to predict diabetic nephropathy via SVM. **Table S12**. Performance of using variable numbers of clinical features to predict diabetic nephropathy via Naive Bayes. Click here for file

Additional file 3 Table S13-S22: **Performance of individual and selected genetic features for diabetic nephropathy classification **This file can be viewed with: **Microsoft Excel Viewer Table S13**. Performance of individual genetic features for diabetic nephropathy classification via decision tree. **Table S14**. Performance of individual genetic features for diabetic nephropathy classification via random forest. **Table S15**. Performance of individual genetic features for diabetic nephropathy classification via SVM. **Table S16**. Performance of individual genetic features for diabetic nephropathy classification via Naïve Bayes. **Table S17 **Ranking of the 20 genetic features based on their information gain scores. **Table S18**. Ranking of the 20 genetic features based on their F-scores. **Table S19**. Performance of using variable numbers of genetic features to predict diabetic nephropathy via decision tree. **Table S20**. Performance of using variable numbers of genetic features to predict diabetic nephropathy among type 2 diabetics via random forest. **Table S21**. Performance of using variable numbers of genetic features to predict diabetic nephropathy via SVM. **Table S22**. Performance of using variable numbers of genetic features to predict diabetic nephropathy via Naive Bayes. Click here for file

Additional file 4 Table S23-S28: **Performance of utilizing clincal and genetic features for diabetic nephropathy classification**. This file can be viewed with: **Microsoft Excel Viewer Table S23**. Ranking of all clinical and genetic features based on their information gain scores. **Table S24**. Ranking of all clinical and genetic features based on their F-scores **Table S25**. Performance of using variable numbers of genetic and clinical features to predict diabetic nephropathy via decision tree. **Table S26**. Performance of using genetic and clinical features to predict diabetic nephropathy via random forest. **Table S27**. Performance of using genetic and clinical features to predict diabetic nephropathy via SVM. **Table S28**. Performance of using genetic and clinical features to predict diabetic nephropathy via Naive Bayes. Click here for file

Additional file 5 Table S29-S31: **Performance of gender-based diabetic nephropathy classification**. This file can be viewed with: **Microsoft Excel ViewerTable S29**. Performance of gender-based random forest classification of diabetic nephropathy in the training/testing dataset. **Table S30**. Performance of gender-based SVM classification of diabetic nephropathy in the training/testing dataset. **Table S31**. Performance of gender-based Naïve Bayes classification of diabetic nephropathy in the training/testing dataset. Click here for file

Additional file 1 TableS 1-S2: **Demographic and clinical characteristics of the participants**. This file can be viewed with: **Microsoft Excel Viewer Table S1**. Demographic and clinical characteristics of the participants. **Table S2**. Genotype distributions of the significant candidate genes in T2D patients with and without diabetic nephropathy. Click here for file

## Declarations

The authors declare that funding for the publication of this manuscript was sponsored by the Ministry of Science and Technology of the Republic of China, Taiwan.

This article has been published as part of *BMC Bioinformatics *Volume 16 Supplement 1, 2015: Selected articles from the Thirteenth Asia Pacific Bioinformatics Conference (APBC 2015): Bioinformatics. The full contents of the supplement are available online at http://www.biomedcentral.com/bmcbioinformatics/supplements/16/S1
